# Inverse kinematics solution and control method of 6-degree-of-freedom manipulator based on deep reinforcement learning

**DOI:** 10.1038/s41598-024-62948-6

**Published:** 2024-05-30

**Authors:** Chengyi Zhao, Yimin Wei, Junfeng Xiao, Yong Sun, Dongxing Zhang, Qiuquan Guo, Jun Yang

**Affiliations:** University of Electronic Science and Technology of China, Shenzhen Institute for Advanced Study, Shenzhen, 518110 China

**Keywords:** Intelligent manufacturing, Manipulators, Inverse kinematics solution, Artificial intelligence, Digital twin, Reinforcement learning, Electrical and electronic engineering, Mechanical engineering

## Abstract

The advent of Industry 4.0 has significantly promoted the field of intelligent manufacturing, which is facilitated by the development of new technologies are emerging. Robot technology and robot intelligence methods have rapidly developed and been widely applied. Manipulators are widely used in industry, and their control is a crucial research topic. The inverse kinematics solution of manipulators is an important part of manipulator control, which calculates the joint angles required for the end effector to reach a desired position and posture. Traditional inverse kinematics solution algorithms often face the problem of insufficient generalization, and iterative methods have challenges such as large computation and long solution time. This paper proposes a reinforcement learning-based inverse kinematics solution algorithm, called the MAPPO-IK algorithm. The algorithm trains the manipulator agent using the MAPPO algorithm and calculates the difference between the end effector state of the manipulator and the target posture in real-time by designing a reward mechanism, while considering Gaussian distance and cosine distance. Through experimental comparative analysis, the feasibility, computational efficiency, and superiority of this reinforcement learning algorithm are verified. Compared with traditional inverse kinematics solution algorithms, this method has good generalization and supports real-time computation, and the obtained result is a unique solution. Reinforcement learning algorithms have better adaptability to complex environments and can handle different sudden situations in different environments. This algorithm also has the advantages of path planning, intelligent obstacle avoidance, and other advantages in dynamically processing complex environmental scenes.

## Introduction

With the rapid development of emerging technologies such as big data, artificial intelligence, and cloud computing, robotics and intelligent methods have been rapidly developed and widely applied in the context of Industry 4.0. In industrial production, the control of manipulators is gradually shifting from manual teaching to motion control algorithms. The use of forward and inverse kinematics algorithms can achieve intelligent control of manipulators. Therefore, the study of motion control algorithms for manipulators is of great significance to industrial production. Six-degree-of-freedom manipulators are widely used in industrial production, such as mechanical assembly, welding, handling, grasping, and disassembly^1^. These applications often require high-precision manipulator control, and the operating environment is complex and diverse. Traditional manipulator control algorithms are insufficient in complex environments and scenarios with sudden changes. Therefore, it is necessary to improve the motion control algorithm for manipulators^1^.

The goal of the inverse kinematics problem for robotic arms is to determine the angle of each joint given the position and posture of the end effector. The traditional inverse kinematics solution methods of manipulators mainly include algebraic methods^4^, geometric methods^5^ and numerical methods^6^. Algebraic methods obtain the kinematic equations through strict coordinate transformations. Algebraic methods have high accuracy and fast speed in computation, but there is no universal method for solving nonlinear equations. The geometric method is mainly applicable to special configuration robotic arms that comply with the Pieper criterion, and is solved by adding constraints. The calculation amount is generally smaller than analytical and numerical methods, but the universality is far inferior to algebraic methods. Numerical methods solve the kinematic equations by iterative methods, including the Newton–Raphson iteration^7^, pseudo-inverse iteration^8^, gradient projection^9^, etc. And numerical methods are feasible in most cases, but they cannot obtain all solutions. The calculation amount is very large and requires repeated iterations, which is not suitable for real-time computing tasks. Doty et al.^10^ analysed 6-DOF industrial robots’ framework. Ozgoren et al.^11^ used analytical methods to solve the inverse kinematics of redundant manipulators.

To overcome the limitations of traditional methods, some intelligent algorithms are used in solving the inverse kinematics of robotic arms, such as heuristic algorithms, neural network algorithms, and other machine learning methods. Heuristic algorithms include genetic algorithms(GA)^12^, particle swarm optimization(PSO)^13^, ant colony optimization(ACO)^14^, Vortex Search^15^, etc., which draw inspiration from intelligent behaviors in nature or biological systems. Heuristic algorithms employ heuristic strategies like random search, population evolution, etc., to simulate these processes and solve inverse kinematics problems. Mustafa et al.^16^ developed a 4-DOF serial robotic manipulator and used genetic algorithms (GA), particle swarm optimization (PSO), quantum particle swarm optimization (QPSO)^17^, and gravitational search algorithm (GSA)^18^ to solve its kinematics inverse. Liu et al.^19^ proposed a universal robotic inverse kinematics solution method based on improved PSO algorithm, aiming to overcome issues like robustness and local optimums in the particle swarm algorithm. Heuristic algorithms, unlike algebraic methods, do not require precise mathematical models, are generally robust, and can handle various constraints. However, these algorithms may be sensitive to parameter selection and prone to local optimum problems. Some researchers have used neural network to solve the inverse kinematics of manipulators^20–25^. Demby et al.^21^ used neural networks and adaptive fuzzy inference recommendation systems to solve the inverse kinematics of manipulators with different degrees of freedom. Bai Y et al.^22^ used the FOA optimized BP neural network algorithm to solve the robot kinematics can improve the control accuracy of the robot. Shiping et al.^25^ proposed a method based on convolutional neural network models to solve the inverse kinematics of redundant manipulators and conducted trajectory tracking experiments to verify that convolutional neural networks can solve kinematic inverse solutions with high accuracy. These methods utilize advances in computing power and deep learning algorithms to attempt to address some of the challenges encountered by traditional methods. But in some cases, a large number of training samples or parameter adjustments are still needed to achieve good performance.

In recent years, due to the generalization ability of reinforcement learning algorithms, using them to solve inverse kinematics problems of robotic arms has become a popular direction^26–28^. Reinforcement learning is a field of machine learning that discovers optimal behavior policies through trial and error^29,30^. Reinforcement learning learns the mapping from environmental states to actions, with the goal of maximizing the cumulative reward value obtained by actions from the environment^2^. Common algorithms include value function methods such as Q-learning, SARSA, DQN, and policy gradient methods such as REINFORCE, TRPO, PPO, etc. Some researchers have studied the use of reinforcement learning to control a multi axis manipulator^3,31–37^. Zichang et al.^33^ use DDPG algorithm to obtain the inverse kinematics of 5-DOF arm robot. Malik et al.^2^ use PoE as a Forward Kinematics computation tool and the DQN as an IK solver. Adolfo et al.^35^ regard each joint of the robot as one agent to train a reinforcement learning model. Zhang et al.^37^ proposed an improved PPO algorithm that improved both the convergence speed and the operating accuracy. The value function method is not friendly to the continuous action space, while the strategy gradient method can directly optimize the continuous control strategy, which is more suitable for inverse kinematics problems of robotic arms. The PPO algorithm controls the amplitude of policy updates through clipping techniques, making training more stable and computing more efficient. It combines the advantages of strategy gradient methods, which can handle high-dimensional continuous action spaces while ensuring the stability of the learning process. Previous studies have shown that PPO can quickly and efficiently learn strategies for accurately mapping the angles of each joint of a robotic arm. At present, methods that use reinforcement learning algorithms to control robotic arms typically perform well in optimizing position control, but do not fully consider the accuracy of posture, such as the study by Malik et al.^2^. However, the algorithm proposed in this paper not only focuses on optimizing position control, but also on posture control of the end effector of the robotic arm. This method simultaneously considers and optimizes posture information during the control process.

This paper proposes a six-degree-of-freedom manipulator inverse kinematics solution method based on PPO algorithm. Using the Unity development engine, a digital twin simulation environment is created for the manipulator. Reward and punishment functions, environmental perception parameters, and target update strategies are designed in the simulation environment. Multi-agent parallel training method is used for reinforcement learning training to obtain the manipulator's inverse kinematics solution and motion control strategy. The reinforcement learning method proposed in this paper overcomes the multiple solution problem faced by traditional methods in singular positions of manipulators. This method uses multi-agent method to obtain faster training speed and computational efficiency than one agent method. Unlike the slow calculation speed caused by repeated iterations of iterative methods, this method supports real-time calculation of inverse solutions and motion strategies. Traditional inverse kinematics solution methods require the development of motion planning algorithms to achieve manipulator control, and must be pre-set for fixed reactions for fixed scenes. This paper uses reinforcement learning training to calculate motion strategies in real-time, which is suitable for more complex and changeable environments. The experimental results show that the results obtained by this method are relatively accurate, and the calculation results are effective single solutions with continuous and smooth joint angle changes, which meet the requirements of motion.

##   The kinematic model and simulation environment

### Kinematic model of robotic arm

The kinematic model of the robot is built by the DH convention. We use this model to solve the inverse kinematics and calculate the joint angles required to achieve desired end-effector positions and poses. The DH parameter table of the manipulator configuration is shown in Table 1.

**Table 1:** The DH parameter table of the manipulator configuration.

i	α	a	d	θ
1	0	0	0	θ_1_
2	− 90°	0	0	θ_2_
3	0	a_2_	d_3_	θ_3_
4	− 90°	a_3_	d_4_	θ_4_
5	90°	0	0	θ_5_
6	− 90°	0	0	θ_6_

We use rotation matrix to transform coordinates from the base coordinate system of the robotic arm to the end-effector coordinate system. This transformation method will aid in kinematic analysis. The rotation matrix formula of the computer arm end attitude is as follows: (1)

1 $$\begin{array}{*{20}c} {{}_{i}^{i - 1} T = \left( {\begin{array}{*{20}l} {\cos \theta_{i} } \hfill & { - \sin \theta_{i} } \hfill & 0 \hfill & {a_{i - 1} } \hfill \\ {\sin \theta_{i} \cos \alpha_{i - 1} } \hfill & {\cos \theta_{i} \cos \alpha_{i - 1} } \hfill & { - \sin \alpha_{i - 1} } \hfill & { - \sin \alpha_{i - 1} d_{i} } \hfill \\ {\sin \theta_{i} \sin \alpha_{i - 1} } \hfill & {\cos \theta_{i} \sin \alpha_{i - 1} } \hfill & {\cos \alpha_{i - 1} } \hfill & {\cos \alpha_{i - 1} d_{i} } \hfill \\ 0 \hfill & 0 \hfill & 0 \hfill & 1 \hfill \\ \end{array} } \right)} \\ \end{array}$$

Each Rotation matrix is initialized according to the angle, and the matrix multiplication results are as follows:

2 $$\begin{array}{c}{}_{1}{}^{0}T{}_{2}{}^{1}T{}_{3}{}^{2}T{}_{4}{}^{3}T{}_{5}{}^{4}T{}_{6}{}^{5}T=\left(\begin{array}{cccc}{r}_{11}& {r}_{12}& {r}_{13}& {p}_{x}\\ {r}_{21}& {r}_{22}& {r}_{23}& {p}_{y}\\ {r}_{31}& {r}_{32}& {r}_{33}& {p}_{z}\\ 0& 0& 0& 1\end{array}\right)\end{array}$$

The information of this matrix can also be derived from the position and posture of the robotic arm's end-effector. Therefore, through this equation, we can study both the forward and inverse kinematics of the robotic arm.

### The simulation environment

**Figure 1:**
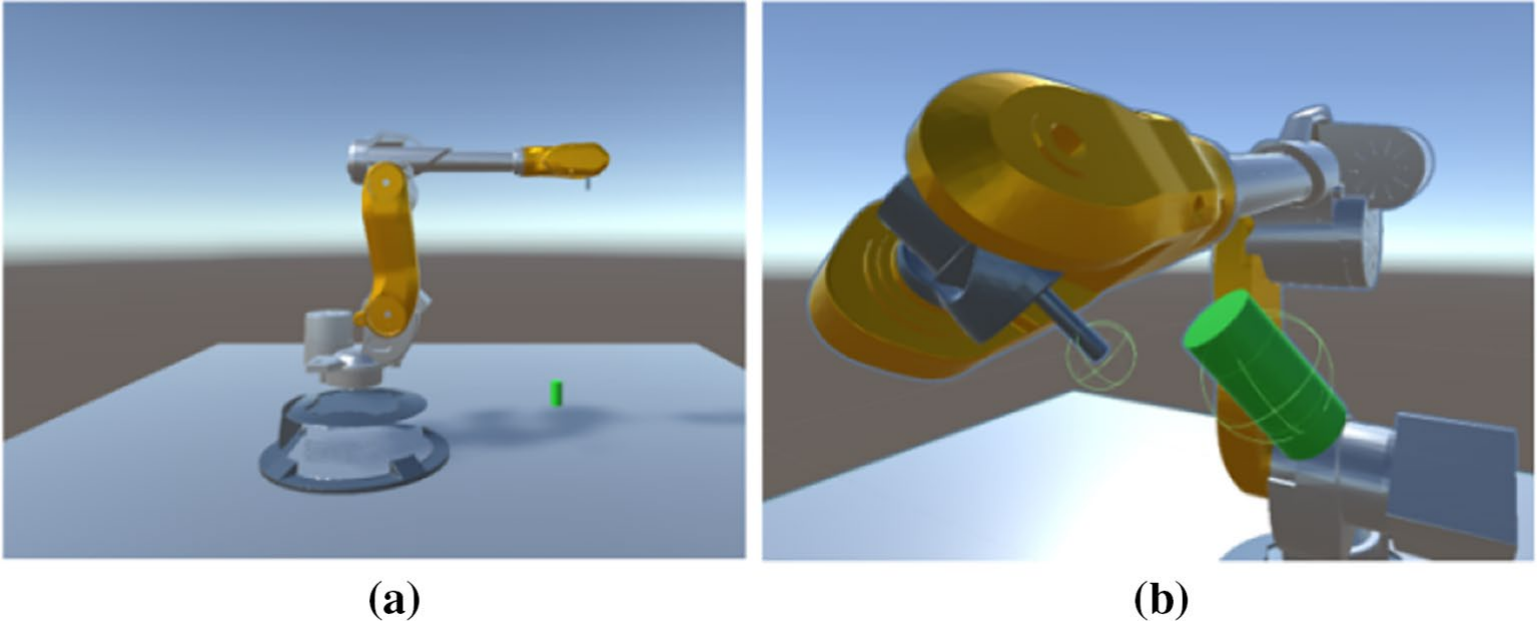
(**a**) Robot arm Reinforcement learning training environment; (**b**) Compare the position and orientation of the end effector of the robotic arm and the target object.

The main body of the reinforcement learning training environment for the manipulator consists of three parts: the manipulator body, the ground, and the target, as shown in Fig. 1. Each agent unit contains a six-degree-of-freedom manipulator, the ground, and a green cylindrical target. This paper uses the Unity development engine to construct the reinforcement learning training environment.

The six-degree-of-freedom manipulator is set to have a range of rotation angles for each of its six joints. The action of the agent is presented by the manipulator body. The central controller obtains the current state information and reward situation, and transmits a six-dimensional decision vector to the manipulator, which decides the changes in the six joint angles of the manipulator. The decision vector is as follows:

3 $$\begin{array}{c}Decision Vector= \left[{\Delta a}_{1},{\Delta a}_{2},{\Delta a}_{3},{\Delta a}_{4},{\Delta a}_{5},{\Delta a}_{6}\right]\end{array}$$

Through the changes in the six joint angles, the end of the manipulator can move to the desired position and posture. The ground supports the manipulator and has obstacle labels and collision bodies. If the end of the manipulator collides with the ground, the agent is punished and the manipulator state is reset. The target represents the target position and posture of the end of the manipulator. If the end of the manipulator moves to the target position and the end posture is consistent with the target, it means that the target has been reached, and a reward is given before resetting the entire environment status for the next learning attempt.

Collision bodies are set for the end of the manipulator, the ground, and the target objects. The collision body settings for the end and the target are shown in the figure. Different reactions occur when collisions occur between them: collision between the end of the manipulator and the target represents approaching the target position, and a reward is given before resetting the environment; collision between the end of the manipulator and the ground represents hitting an obstacle, and the manipulator state is reset after punishment; collision between the target and the ground represents an abnormal update of the target position, and the target state needs to be reset.

## Reinforcement learning algorithm

This paper introduces a deep reinforcement learning method to solve the inverse kinematics and motion control of the six-degree-of-freedom manipulator and proposes a reinforcement learning method based on the MAPPO algorithm. By setting the reward and punishment function and training the agent, it can learn autonomously and complete the inverse kinematics solution and motion planning autonomously.

### Reinforcement learning framework

**Figure 2:**
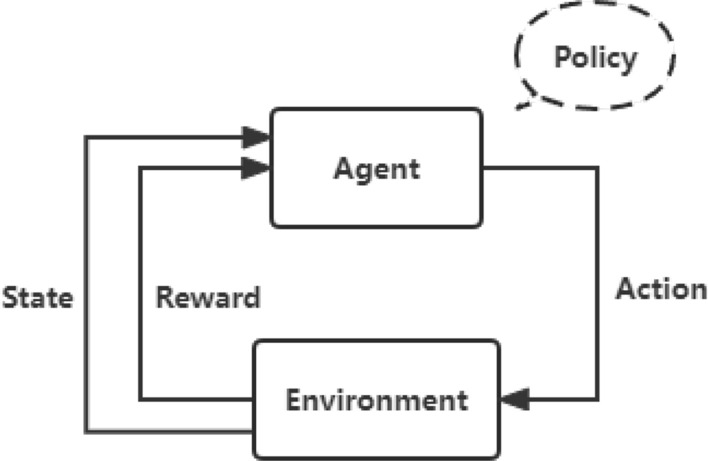
Schematic diagram of reinforcement learning.

The reinforcement learning framework, as shown in Fig. 2, usually includes six basic elements: environment, agent, state, action, reward, and policy^11^. The relationship between them is shown in the figure. The agent is the entity that learns the policy. It will execute actions based on the current state and obtain rewards from the environment. The environment represents the space environment that the agent wants to explore. It will respond according to the agent's actions and give rewards. The reward can be positive or negative, representing whether the action has a positive or negative impact on the current goal. The state is a description of the environment, and the agent makes decisions about the next action based on the state. The policy is the method that the agent uses to select actions based on the state, and the policy can be deterministic or stochastic.

### Design of environmental perception parameters

Environmental parameters refer to the input vector of the neural network. In this paper, the perception parameters are designed as a 16-dimensional vector, and the parameters are described as shown in Table 2.

**Table 2:** Environmental perception parameter table.

Parameter	Description
angles[0]	Joint angle of manipulator joint 1
Angles[1]	Joint angle of manipulator joint 2
angles[2]	Joint angle of manipulator joint 3
angles[3]	Joint angle of manipulator joint 4
angles[4]	Joint angle of manipulator joint 5
angles[5]	Joint angle of manipulator joint 6
relativePosition	The relative position of the target object to the end effector of the manipulator, normalized
relativeRotation	The difference between the target object posture vector and the end effector posture vector of the manipulator, normalized
euclidean_distance	The Gaussian distance between the target object and the end effector of the manipulator
cosine_distance	The cosine distance between the target object posture and the end effector posture of the manipulator
final_distance	Describes the difference in position and posture between the target object and the end effector of the manipulator
StepCount	Iteration count

### Reward and punishment function design

When the end effector of the manipulator reaches the target posture, the agent is given a reward based on the difference between the end effector and the target position and posture. The reward calculation formula is as follows:

4 $$\begin{array}{*{20}c} {reward = 2 + 0.006 - distance*1000} \\ \end{array}$$

Here, reward is the reward value, and distance represents the difference between the end effector and the target position and posture. This is done to encourage the manipulator to move towards a position and posture closer to the target.

When the joint angle of the manipulator exceeds the limit range, a punishment of 1.0 is given to the agent, and the joint angles of the manipulator are reset. This is done to limit the manipulator's operation within the normal range.

For each iteration, a punishment of 0.0005 is given to the agent to speed up the manipulator's movement to the target posture and avoid excessive iteration times.

### Training sample update strategy design

To achieve the purpose of computing the inverse kinematics of the manipulator, this paper proposes a target update strategy based on forward kinematics. The position and posture of the target updated each time are solved by the kinematic equations to obtain the new position and posture of the target.

Each update, each joint angle of the manipulator generates a random angle within the limited range. Each rotation matrix is initialized according to the angle. From the fourth columnof matrix in Eq. (2), the target position can be obtained, and the target posture can be obtained from the 3 × 3 matrix in the upper left corner. After obtaining the position and posture of the target to be updated, the target is moved to the corresponding position and posture, waiting for the manipulator agent to learn to move closer. With this target update strategy, the agent will learn in each iteration how to decide the corresponding angle of each joint movement based on the position and posture of the end effector.

### Virtual agent training algorithms

(1) PPO Algorithm

PPO is a reinforcement learning method^9^. It is based on a method called "policy gradient" to train the agent. This method adjusts the parameters of the agent to change its behavior and maximize the long-term return. One advantage of the PPO algorithm is that it can effectively handle continuous action spaces and high-dimensional state spaces, making it very suitable for complex tasks in many practical applications.

(2) MAPPO Algorithm

**Figure 3:**
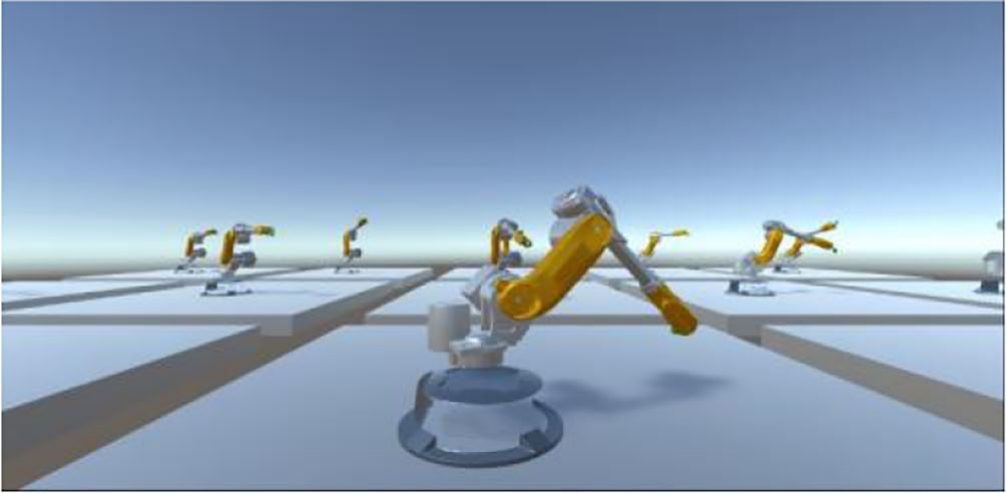
Multi agent reinforcement learning training.

The model in this paper is based on the multi-agent PPO algorithm, an extension of the PPO algorithm^10^, with improved network structure. MAPPO is used to train multi-agent systems and can effectively improve training speed and computational efficiency. The MAPPO algorithm trains multiple agents in parallel simultaneously, which has higher training efficiency than the training method of using a single agent in PPO. And we aim to achieve higher computational efficiency through training optimization. During the training process, the MAPPO algorithm adjusts the parameters of each agent to change their behavior and maximize their long-term return. The multi-agent reinforcement training scenario of MAPPO is shown in Fig. 3.

Each agent in each training scene corresponds to a policy-value network. During the training process, each agent learns to execute its own task independently. The controller obtains the actions, observations, and set rewards of each agent during the training process, and each agent executes its own policy.

This paper proposed a one-step training strategy. By designing the reward function, the difference between the end effector state of the manipulator and the target posture is calculated in real-time. A new type of reward mechanism is designed to reward the behavior of the manipulator approaching the target posture. In this training, the reward function simultaneously considers Gaussian distance and cosine distance, and the overall difference calculation function is as follows:

5 $$\begin{array}{c}distance= euclidean\_distance *cosine\_distance+b\end{array}$$

6 $$\begin{array}{c}b=cosine\_distance-1\end{array}$$

Here, euclidean_distance is the Gaussian distance between the end effector and the target, representing the difference in position between the end effector and the target. cosine_distance is calculated based on the cosine distance between the end effector and the target, with a value range of [1,3], representing the difference in position and posture between the end effector and the target. The product of the two distances represents the overall difference between the end effector and the target, and the constant term b is an offset value with a value range of [0, 2], preventing the loss of posture information caused by the small Gaussian distance. This overall difference calculation function can well obtain the position and posture difference information between the end effector and the target object. For simplicity, based on the consideration of using reinforcement learning to calculate the inverse kinematics in this method, it can be represented as the MAPPO-IK algorithm. The overall process of using the MAPPO-IK algorithm to train the inverse kinematics of the manipulator is shown in Algorithm 1 below:

**Algorithm 1:**
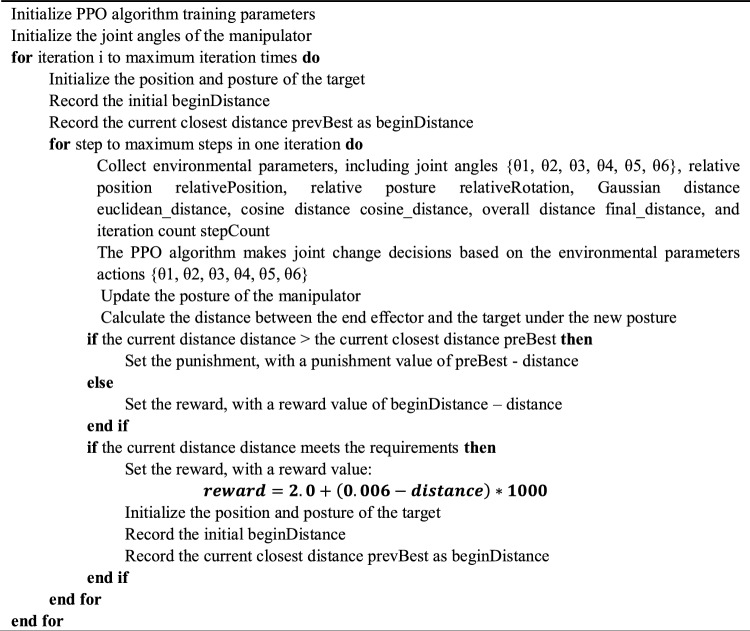
Pseudocode of MAPPO IK for Solving Robot Inverse kinematics

This study also tested another two-step training method for the configuration of the manipulator with the last three axes intersecting. The two-step training method can be used, where the first step trains the end effector to approach the intermediate point in the first three axes, and the second step trains the end effector to approach the virtual target point in the last two axes. After reaching the target position, the sixth axis is controlled to adjust the posture angle. The training of the first five axes can be completed in the first stage, and the fine-tuning of posture belongs to the second stage. This algorithm simplifies the training process that simultaneously considers the displacement difference and rotation difference to first consider the displacement and then consider the posture. The two stages focus on one training task, thus obtaining more accurate training results. However, this method also needs to consider the manipulator configuration. Although it does not require deriving the inverse kinematics solution algorithm for the manipulator like traditional algorithms, it still needs to adjust the reinforcement learning training algorithm based on the manipulator configuration. The generalization is not as good as the one-step training algorithm. Therefore, this paper proposed a one-step algorithm, and all experimental results were obtained from the one-step algorithm.

## Model training and result analysis

### Training parameters

The reinforcement learning training parameters are set as shown in Table 3.

**Table 3:** Reinforcement learning training parameters.

Parameter	Default value	Value used
trainer_type	ppo	ppo
batch_size	256	1024
buffer_size	2560	20,480
learning_rate	3.0e-4	2.0e-4
hidden_units	256	512
num_layers	3	3
vis_encode_type	simple	resnet
max_steps	10.e6	30.0e6
time_horizon	64	512

### Training results

**Figure 4:**
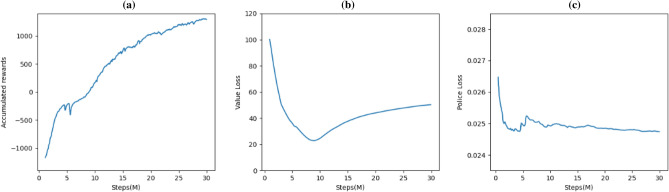
MAPPO-IK algorithm training results: (**a**) accumulated rewards; (**b**) value loss; (**c**) policy loss.

The training model is evaluated based on training loss, cumulative reward, and policy loss. The training results are shown in Fig. 4. The cumulative reward is the average cumulative reward obtained by training the manipulator agent using the MAPPO-IK reinforcement learning algorithm. From the figure, it can be seen that with the increase of the iteration times, the cumulative reward obtained by the MAPPO-IK algorithm gradually increases and tends to stabilize, reaching a converged state. The loss value represents the difference between the model result and the target result. The smaller the loss value, the smaller the difference between the position and posture of the end effector obtained and the target. From the figure, it can be seen that with the increase of the iteration times, the loss also decreases.

**Figure 5:**
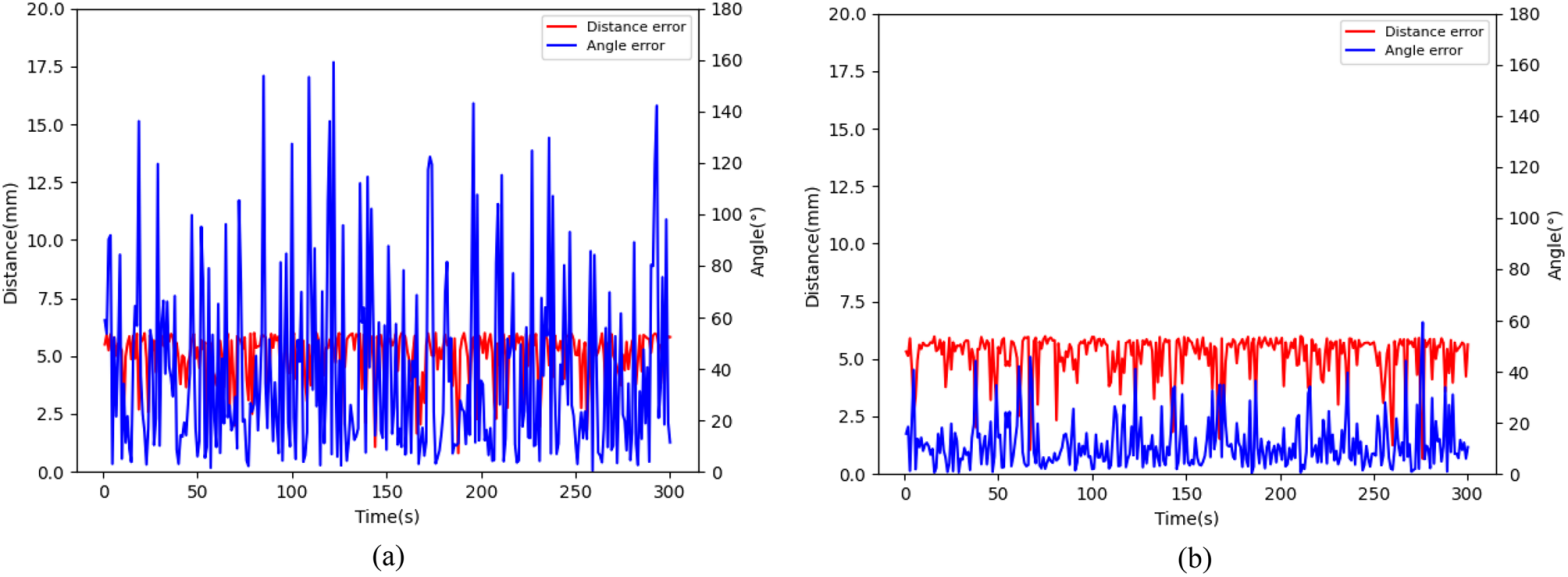
Distance error and angle arror results using different algorithm: (**a**) MAPPO; (**b**) MAPPO-IK.

To verify the effectiveness and accuracy of the MAPPO-IK algorithm in solving the inverse kinematics, the training model is tested by randomly generating target points. The distance between the end effector and the target, as well as the angle between the end effector vector and the target vector, are used to evaluate the position and posture difference between the end effector and the target. The position and posture of the target are random. The test results are shown in Fig. 5.

From the figure, it can be seen that the displacement error is between 0-6mm and the angle error is basically below 10 degrees when using the MAPPO-IK algorithm. Obviously, the results obtained using the traditional MAPPO algorithm have significant angle errors, which means that the posture of the end effector of the robotic arm is completely different from the target.

**Figure 6:**
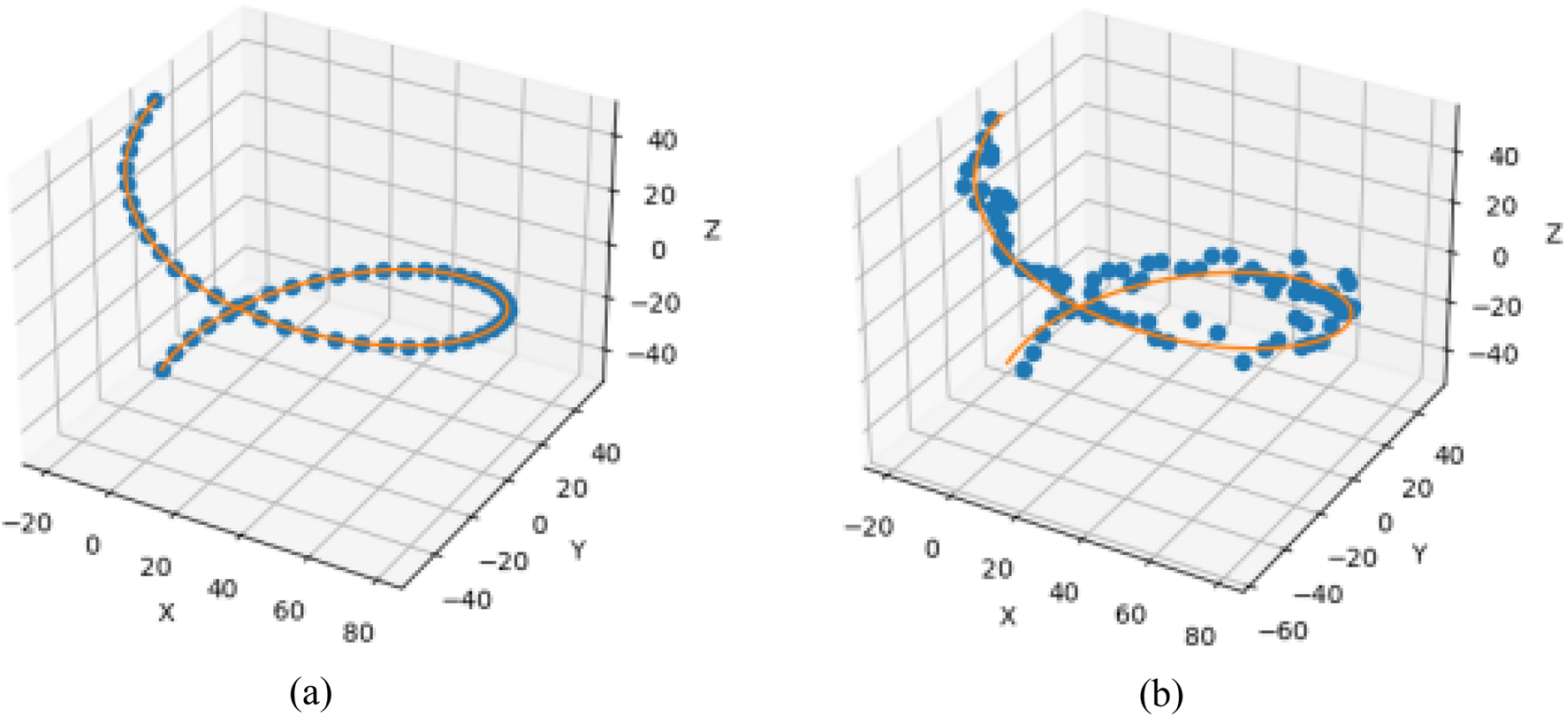
MAPPO-IK algorithm for continuous motion trajectory fitting: (**a**) expected trajectory; (**b**) actual trajectory.

To verify the effectiveness of the manipulator control using the trained model, the manipulator is controlled to perform continuous motion using the reinforcement learning model based on the given expected trajectory. As shown in Fig. 6, the path obtained based on reinforcement learning is almost identical to the expected path.

**Figure 7:**
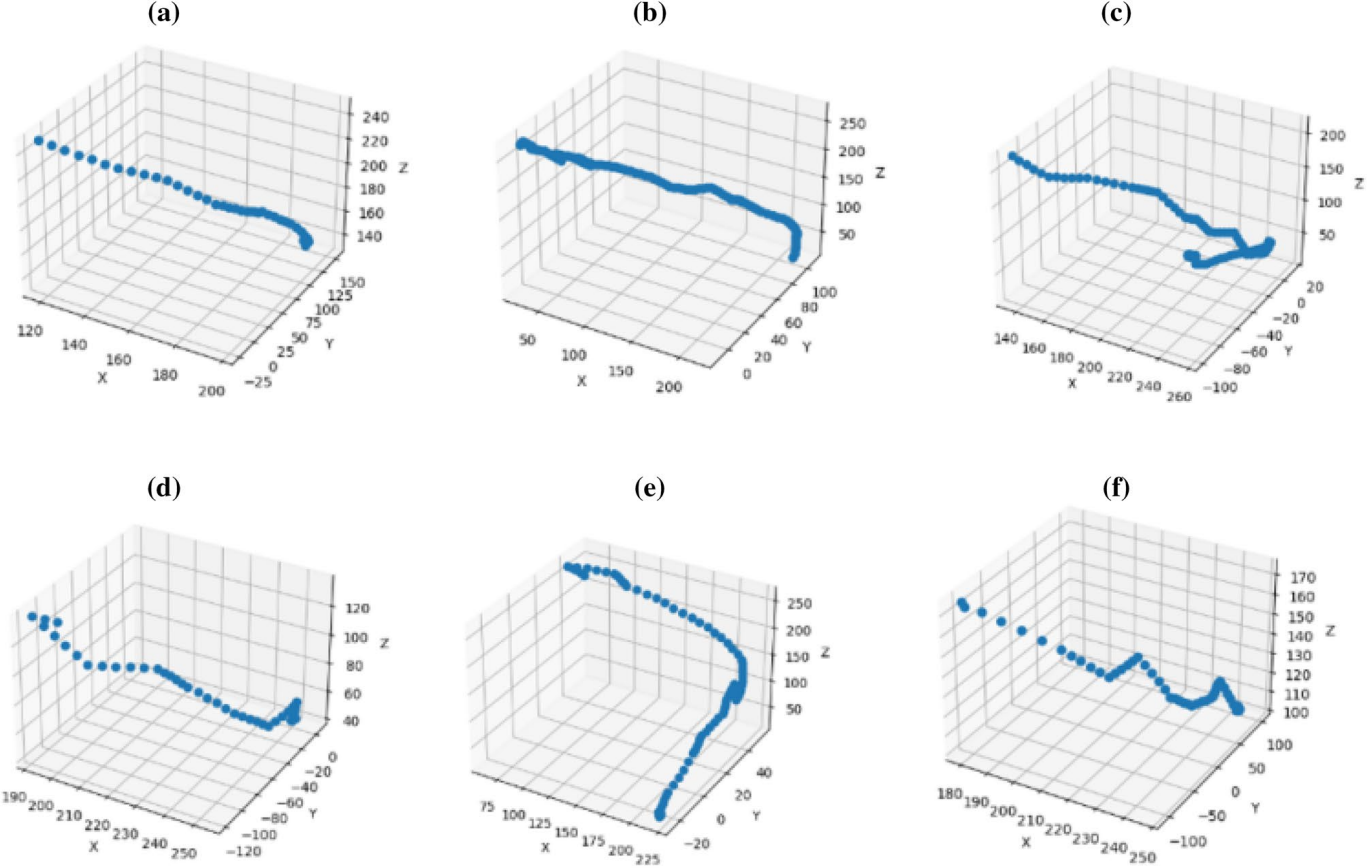
Different path plans obtained from the MAPPO-IK algorithm.

To verify the path planning ability of the MAPPO-IK model, six sets of different point pairs are designed, each set containing the position and posture information of two target points. The MAPPO-IK algorithm will control the end effector of the robotic arm to move from A to B. The experiment records the path of the end effector. As results shown in Fig. 7, the continuous smooth curves indicate that the MAPPO-IK model has the ability to control and plan paths for the manipulator.

This paper uses a convolutional neural network to train the model and compares it with the same dataset. The convolutional neural network is a deep learning method, which can also be used to train the inverse kinematics of the manipulator. It is a universal computing method, which is better than traditional inverse kinematics solution algorithms in terms of generalization because it does not require derivation. The principle of the convolutional neural network algorithm for solving the inverse kinematics of the manipulator is to use a transformation matrix to describe the posture of the manipulator, and the rotation matrix is multiplied in turn to obtain the posture description matrix of the end effector of the manipulator, as described in Eq. (2). The first three rows are taken as the input of the model, that is, the model input is a 3*4 matrix, and the output is a 6-dimensional vector, with each of the 6 elements corresponding to the joint angles of the manipulator. The convolutional neural network uses a convolutional kernel to perform convolution operations on the input matrix to extract the posture features of the manipulator. The CNN model we used for comparative experiments was based on the research of Shiping et al.^13^. This paper used a convolutional neural network with consistent parameters and also employed L2 norm regularization optimization method.

**Figure 8:**
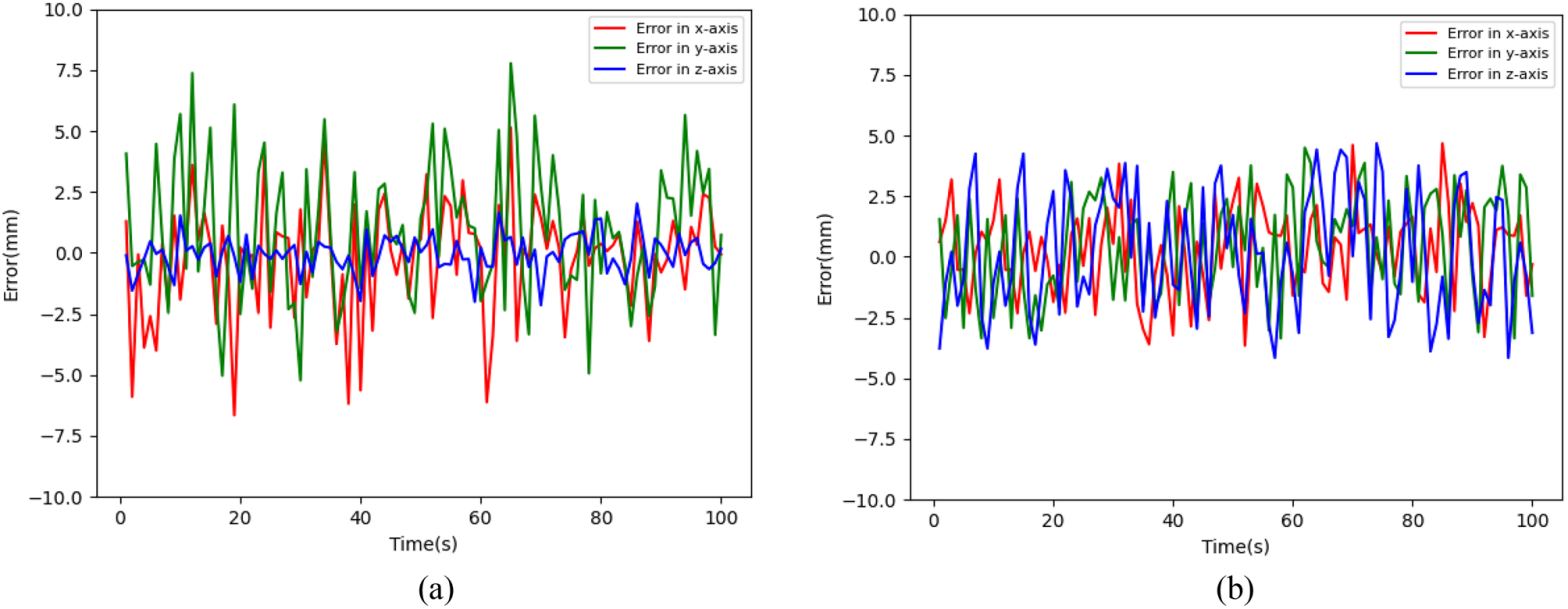
Error comparison between MAPPO-IK algorithm and CNN algorithm: (**a**) CNN; (**b**) MAPPO-IK.

This paper choose the error of the Cartesian error as the comparison object, and the results are shown in Fig. 8.

Through the experimental results comparison, we can know that the MAPPO-IK algorithm for calculating the inverse kinematics is superior to other inverse kinematics calculation methods in terms of position control accuracy. The accuracy of the end effector’s position of MAPPO-IK is significantly better than another one. It is demonstrated that the Cartesian error of MAPPO-IK stays within the bounds of ± 4 mm and that of CNN stays within the bounds of ± 6 mm. In addition, the MAPPO-IK algorithm has the advantages of path planning and intelligent obstacle avoidance in dynamically processing complex environmental scenes, while the convolutional neural network only supports static inverse kinematics solution.

## Experimental verification

To verify the superiority of reinforcement learning in manipulator control and practical scenarios, a virtual experiment platform and a virtual-real synchronous digital twin experiment platform in real scenes were built.

**Figure 9:**
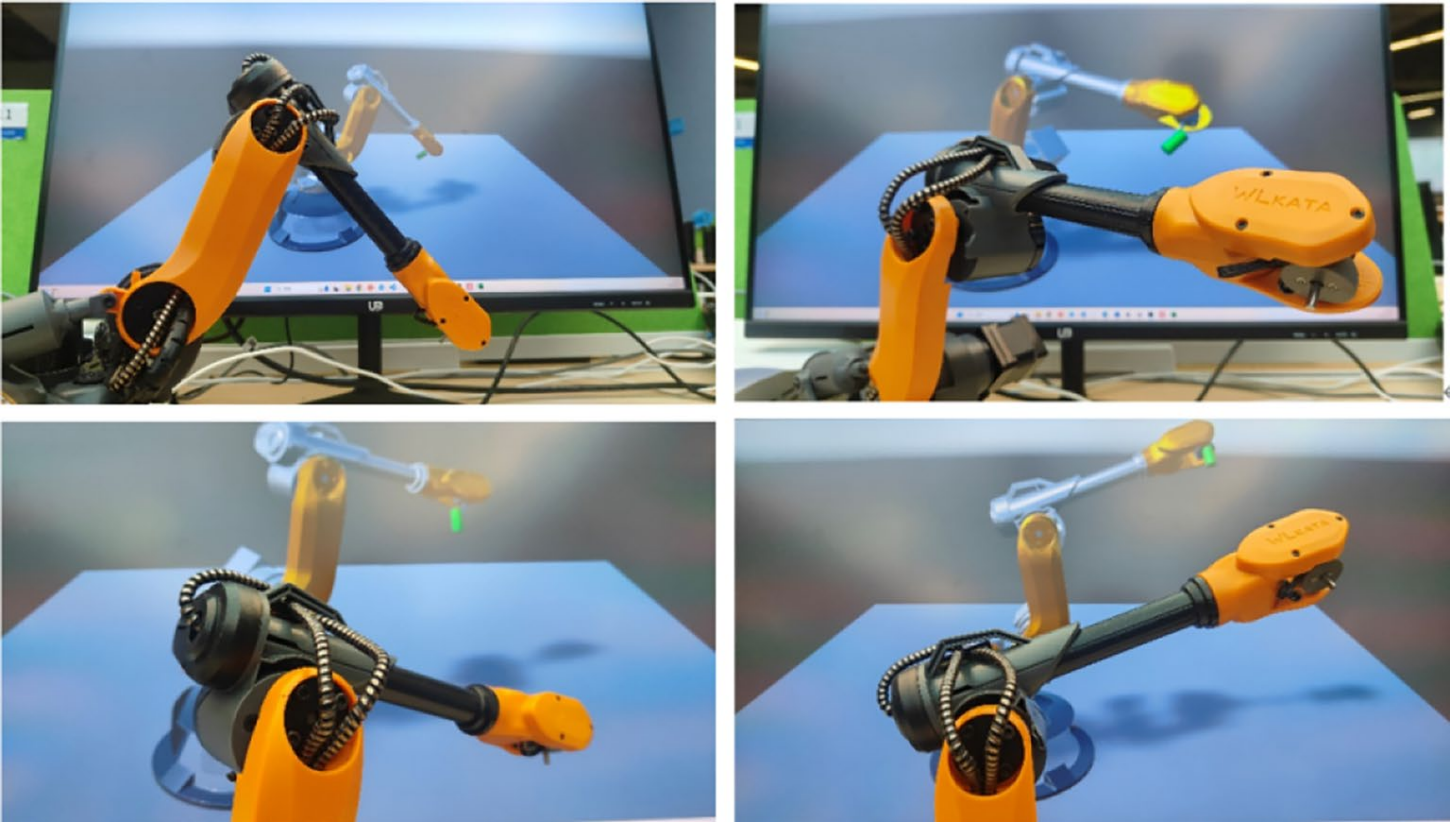
Virtual real synchronous digital twin system.

In the virtual environment, the manipulator will approach the target in position and posture. Through the virtual experiment platform, we can intuitively observe the superiority of the reinforcement learning algorithm. The same test is also conducted in the virtual-real synchronous digital twin experiment platform, where the digital twin of the manipulator in the virtual space can control the physical manipulator synchronously. This control method has huge potential and can collect environmental information through external devices such as cameras and sensors. The MAPPO-IK model makes decisions such as path planning and intelligent obstacle avoidance in the virtual space based on environmental information, and is deployed in the physical manipulator. The virtual-real synchronous digital twin system is shown in Fig. 9.

## Conclusion

In this work, it was shown that the Deep Reinforcement Learning approach is viable for solving the Inverse Kinematics problem of a 6-DOF robotic manipulator. Traditional methods for calculating the inverse kinematics of manipulators often face the problem of insufficient generalization. Geometric methods require special manipulator configurations, algebraic methods require different algebraic solution methods to be derived for different manipulator configurations, and traditional calculation methods often face the problem of multiple solutions at singular positions. Iterative methods face challenges such as large computation and long solution time. The neural network-based solution method often requires a large amount of data as training support. This paper proposes a reinforcement learning-based inverse kinematics solution algorithm, called the MAPPO-IK algorithm. The reinforcement learning method proposed in this paper overcomes the shortcomings of traditional methods and can learn autonomously based on the environment, without requiring a large amount of training data. Compared with traditional inverse kinematics solution algorithms, this method has good generalization and supports real-time computation, and the obtained result is unique. The angle accuracy of MAPPO-IK algorithm is significantly better than that of convolutional neural networks, which also have good generalization. Reinforcement learning algorithms have better adaptability to complex environments and can handle different sudden situations in different environments. Therefore, this inverse kinematics solution algorithm has the potential to intelligently avoid obstacles while calculating the inverse kinematics and motion planning. Through experimental comparative analysis and physical manipulator experiments, this paper verifies the feasibility, computational efficiency, and superiority of this reinforcement learning algorithm.

## Data Availability

The datasets used and/or analysed during the current study available from the corresponding author on reasonable request.
